# Political ecology: past, present, and future

**DOI:** 10.1007/s43621-026-02707-x

**Published:** 2026-02-07

**Authors:** Ishfaq Hussain Malik, Radhika Borde, James D. Ford

**Affiliations:** 1 School of Geography, University of Leeds, Leeds, UK; 2 Priestley Centre for Climate Futures, University of Leeds, Leeds, UK

**Keywords:** Political ecology, Power dynamics, Environmental justice, Colonialism, Climate change, Decolonisation, Intersectionality

## Abstract

Political ecology examines the interconnectedness of social, political, and ecological processes, offering critical insights into power dynamics, environmental governance, justice, and inequality. We examine the discipline of political ecology and its relevance in understanding the impacts of environmental changes, economic and colonial exploitation, and socioeconomic inequalities. We present an in-depth critical analysis of key debates and themes in contemporary political ecology: decolonial approaches and inclusion of Indigenous knowledge, climate justice and uneven distribution of climate vulnerabilities, posthumanism and more-than-human governance, and Anthropocene and Capitalocene. This paper discusses the economic drivers and structural solutions to climate adaptation. By highlighting the interdisciplinary nature of political ecology, this study illustrates how recent advancements in the field contribute to the development of more equitable environmental governance and global sustainability initiatives. The study discusses the implications and future directions of political ecology, emphasising the need to decolonise the field, address intersecting social categories, apply Indigenous knowledge and knowledge co-production, engage with environmental justice movements, and critically examine AI-mediated climate governance and decision-making.

## Introduction

Political ecology begins with a simple but urgent insight: environmental change is never just environmental—it is political, historical, and profoundly unequal. Political ecology is the study of the political, economic, and social drivers of environmental change and the ways these forces unevenly distribute environmental benefits and burdens [1–3]. It seeks to answer core questions such as: *How do changes in the environment impact different sections of society and regions globally? What are the potential inequalities and power dynamics that arise from the differential effects of environmental changes and exploitation on society? How does the historical legacy of colonialism continue to shape contemporary vulnerability, social dynamics, access to, and control over natural resources?* It is therefore clear that political ecology is concerned with issues of distributive justice (the principle concerned with how risks, harms, and benefits are allocated across societies) as well as the politics of recognition [4–6]. More recently, moving away from the anthropocentrism with which it has been charged [7], it is asking: *How is non-human nature differentially impacted by environmental changes and by environmental policy changes? Can we examine the politics of human-wildlife conflicts from the perspective of wildlife, as well as differentially positioned humans?*

Political ecology is an interdisciplinary field that examines the complex and dynamic relationship between the environment and society, focussing on power dynamics, conflicts, and inequalities in environmental decision-making [8]. It interrogates how environmental processes and resource access are shaped by structural inequalities, historical legacies, and contested governance arrangements [9]. Political ecology compels us to recognise that today’s environmental crises unfold through histories of colonialism, capitalism, and structural inequality—and cannot be understood apart from them [10, 11].

Recent global events and crises have exposed the political and ecological fragility of our current moment. Floods in Dubai and Sharjah, the devastation of Hurricane Maria in Puerto Rico, water shortages in South Africa, intensifying heat waves, student climate strikes, biodiversity crisis, and widespread wildfires all demonstrate how unsustainable existing systems have become. These pressures are unfolding alongside a global cost-of-living crisis and the escalating impacts of climate change [12–14]. This framing positions climate change not merely as an environmental phenomenon, but as a socio-political process shaped by legacies of colonialism, global capitalism, and unequal power relations [15, 16]. The inequalities, injustice, environmental challenges, and conflicts over resources that have arisen because of economic growth and power imbalances make political ecology more relevant than ever [17].

In recent years, global crises such as extreme heatwaves, floods, and biodiversity loss—coupled with the rise of climate justice movements and renewed interest in decolonial approaches—have propelled political ecology into critical interdisciplinary debates [12, 14]. The COVID-19 pandemic further underscored the intricate linkages between ecological change, social vulnerabilities, and governance failures [18]. As a result, the field has expanded to engage with diverse perspectives, including Indigenous knowledge systems, post-humanist ethics, and critiques of green colonialism [19, 20].

Little did Frank Thone know that the term “political ecology,” which he coined in 1935, would lead to almost a century of conceptual debate [21]. Eric Wolf gave it a new life in 1972 in an article entitled “Ownership and Political Ecology," in which he discussed the relationship between ownership and political ecology in the Alps, emphasising the need to understand how ecological changes affect resource distribution and ownership dynamics within households [22]. However, broadly, the concept of political ecology can be traced back to Karl Marx and Friedrich Engels, who argued in the historical dialectical materialism that humans act as a force of nature and adapt natural resources for their own needs. Through this process, humans not only change the external world but also transform themselves [23]. It primarily emerged and developed within colonial or settler colonial contexts, examining environment-society relations and struggles over access to natural resources [20].

Political ecology can also be linked to the social cooperative anarchism of Peter Kropotkin, who emphasised the importance of mutual aid in evolution and survival, countering the ideas of social Darwinism [24]. This approach bears resemblance to present-day political ecology in terms of its emphasis on production, consideration for marginalised and disenfranchised populations, exploration of local environmental knowledge, and focus on the landscape as a subject of inquiry [24, 25]. The foundation of political ecology also lies in the research of Julian Steward. Steward developed a research framework called “cultural ecology” in the 1920s, which explained human social organisation as a functional adaptation to local environments and argued that the human relationship to the environment was more important in structuring cultural patterns than diffusion from other societies [26, 27].

Political ecology started becoming prominent in the 1960s within the context of global neo-liberalisation and the impact of exogenous forces, such as international development and economic modernization, on local communities and environments in the “Global South” [1, 28]. The emerging prominence of political ecology in the 1960s and 1970s was triggered by the global environmental crisis. Murray Bookchin, Eric Wolf, Hans Magnus Enzensberger, and André Gorz, among others, played pioneering roles in this field. Bookchin published “Our Synthetic Environment” in 1962, Enzensberger wrote an influential article “A Critique of Political Ecology” in 1974, and Gorz published “Écologie et liberté” in 1977 [29–32].

The field’s modern foundations are widely associated with Blaikie and Brookfield’s [1] Land Degradation and Society, which combined political economy with ecological analysis to explain land degradation in the “Global South”. This work remains a cornerstone in showing how environmental change and degradation cannot be separated from questions of power, land tenure, and global political economy. Bryant and Bailey’s [33] contributions on “Third World Political Ecology” highlighted early engagements with environmental justice beyond the Global North, anticipating many concerns that now sit at the heart of climate justice scholarship. While the terminology of climate justice was not used at the time, these contributions revealed the structural inequalities and geopolitical dimensions of environmental change that resonate with contemporary climate debates.

Despite its prominence, the field has often been critiqued for insufficient engagement with ecological processes. Walker [34] famously asked, *"Political Ecology: Where is the Ecology?"* This review responds to this critique by placing ecological processes at the core of its analysis. By doing so, we aim to bridge the gap between ecological and social dynamics in political ecology scholarship, acknowledging the agency of non-human actors and the biophysical realities shaping socio-environmental interactions. In recent years, there has been a growing interest in exploring the theoretical foundations and applications of political ecology and how they have changed over time [9, 35].

Over the past three decades, political ecology has expanded to engage with decolonial and anti-colonial scholarship, Indigenous knowledge systems, and more-than-human approaches. While recent calls to decolonise political ecology are vital [19], it is important to recognise that scholars such as Arturo Escobar, Andrea Nightingale, Susanna Hecht, and Juanita Sundberg have long produced work that addresses colonial legacies, uneven vulnerabilities, and non-human agency in governance — often before such terms gained prominence [36–39]. Acknowledging this continuity avoids mischaracterising the field as having neglected these concerns entirely and instead highlights the need to deepen and broaden such approaches in the context of the climate emergency.

In this article, we examine three interlinked themes in contemporary political ecology — decolonial approaches and Indigenous knowledge, climate justice and uneven vulnerability, and posthumanism and more-than-human governance — with climate change as the connecting thread. By situating these debates within both historical foundations and contemporary urgencies, we show that decolonising political ecology is inseparable from confronting the climate crisis.

## Methodological approach

This study is based on a structured review of the political ecology literature, designed to map key conceptual debates and identify emerging directions within the field. Relevant publications were identified through keyword searches in Scopus, Google Scholar, and Web of Science using terms such as political ecology, decoloniality, posthumanism, more-than-human, climate justice, vulnerability, and environmental governance. To ensure both breadth and depth, the search strategy combined foundational texts with recent contributions published in the field. Our review of relevant literature was chronological, looking at developments across time.

Selected materials were reviewed iteratively and organised through thematic coding. This process enabled the identification of recurring lines of inquiry—including critiques of coloniality and capitalism, engagements with climate justice, the growing influence of more-than-human and posthumanist approaches, and evolving debates on carbon governance, digital environmental monitoring, and the politics of adaptation. The goal was not to produce an exhaustive inventory, but rather to synthesise representative strands of scholarship that illuminate current trajectories and ongoing tensions in political ecology.

## Contemporary themes and debates in political ecology

Political ecology has evolved into a dynamic field interrogating the power relations embedded in socio-environmental interactions. While its foundational concern with political economy and distributive justice remains central, recent scholarship increasingly recognises that ecological processes are not passive contexts but active forces shaping these interactions [25, 34]. Contemporary political ecology studies how global environmental change, vulnerability, policy responses, experiences, and differential resilience are the result of the nexus of ideas, institutions, and interests that have combined to accumulate power at the expense of people and the planet [25, 40–42]. Political ecology examines accumulation by dispossession by analysing how capitalist practices such as land grabbing and privatisation dispossess Indigenous and marginalised communities and contribute to environmental injustices [43, 44].

### Posthumanism and more-than-human governance

Early political ecology tended to centre human actors in environmental governance. The more-than-human turn challenges this anthropocentrism by recognising the agency of non-human entities — species, ecosystems, technologies — in shaping environmental outcomes [45, 46]. This posthumanist turn reconceptualises nature as an active participant rather than a passive backdrop, aligning with calls to reintegrate ecological processes into political ecology [34].

Seminal contributions by Whatmore [47], Braun [48], Castree [49], Nightingale [50], and Sundberg [39] have shown how relational ontologies disrupt human–nature binaries and reveal new ethical and governance possibilities. Anthropological work by Lyons [51] and De La Cadena [52] expands this by situating more-than-human relationships within decolonial contexts, while Ingold’s [53] writings trace these relational sensibilities to longstanding Indigenous and place-based ontologies.

Posthumanist approaches offer a conceptual basis for interrogating dominant human-centred narratives and for reimagining alternative ethical frameworks within political–ecological debates [54]. This involves decentring of human agency to provide to examine the role of non-human entities—plants, animals, ecosystems, and technologies—in social and environmental systems [55, 56]. Dominant economic systems alienate humans from nature by treating nature as a hub of resources meant to be extracted for profit, affecting the connection between human societies and ecosystems, leading to social dislocation and environmental degradation [46, 57]. Political ecology asserts that this alienation is an important driver of environmental destruction [18]. Political ecology analysis of posthumanism challenges this alienation by rejecting the anthropocentricism that treats nature as a passive backdrop to human activity [45]. It recognises the agency of non-human actorsin shaping environmental outcomes [58]. By decentring human agency, it recognises that the environment is an interconnected system where humans are part of a larger web of relationships [59, 60].

Critiques, notably from Swyngedouw [61, 62], caution against de-centring human agency without recognising that climate injustice remains structured by human political economies. Integrating these insights, a climate justice-oriented more-than-human approach acknowledges ecological agency without obscuring the human systems of extraction, governance, and inequality that drive vulnerability. For example, mangrove restoration in Southeast Asia not only enhances coastal protection but also reflects complex entanglements of ecological succession, local livelihoods, and state adaptation policies [63–65]. Similarly, wolf reintroduction in Yellowstone catalysed ecosystem transformations that reveal how non-human agency shapes both ecological and social adaptation to climate change [66].

More-than-human governance is a shift in the conceptualisation of environmental management and extends this analysis by examining how environmental governance structures can integrate non-human actors [67]. Dominant governance models view nature as a resource to be managed for human benefits and prioritise human interests and needs [68]. While more-than-human governance advocates for the rethinking of governance structures and recognition of the agency of non-human actors to incorporate them in decision-making processes [69, 70].

Incorporation of the agency of non-human actors contests traditional anthropocentric governance frameworks that regard nature as separate from society [46, 71]. This shift is important for environmental governance, advocating for more holistic approaches that acknowledge the interconnectedness of non-human and human systems (Burke and Fishel [72, 73]. Posthumanism in political ecology critiques the dualistic frameworks that separate humans from nature, instead promoting an understanding of environmental governance as inherently relational and dynamic [58, 60].

Drawing on Foucault’s [74] concept of biopower, scholars have analysed how environmental policies govern both human and non-human populations. Foucault’s framing of biopower—centred on the management of life, the optimisation of populations, and the regulatory power exercised through institutions—has been influential in demonstrating how environmental governance operates through techniques of measurement, surveillance, categorisation, and control [75, 76]. In political ecology, this has meant tracing how states and conservation regimes regulate land, species, and ecosystems through biopolitical interventions that privilege certain forms of life while rendering others expendable [77, 78]. These debates suggest that integrating biopower with posthumanist insights enables a more nuanced account of environmental governance—one that recognises both the material agency of the non-human world and the ongoing role of political, economic, and colonial structures in shaping whose lives are valued and whose are rendered vulnerable.

### Decoloniality, Indigenous knowledge, and economic exploitation

Political ecology’s decolonial turn interrogates how colonial legacies continue to marginalise Indigenous and local knowledge systems. Yet, as Escobar [10, 36] and Hecht et al. [37] illustrate, such critiques have long been part of the field, even before “decolonial” became a common term. Rather than portraying these perspectives as absent, it is more accurate to show how they have existed but require deepening and institutional transformation to meet today’s climate challenges.

Robust examples include Nightingale’s [50, 79, 80] work in Nepal linking gendered resource governance to climate vulnerability, Tschakert’s [81, 82] research on participatory adaptation in West Africa, and Gonda’s [83, 84] analysis of how authoritarian environmental governance exacerbates social inequality under climate stress. These works operationalise decolonial political ecology by centring local epistemologies, redistributing decision-making power, and revealing how intersecting oppressions shape adaptive capacity.

Critiques of colonialism, capitalism, and the epistemic dominance of Western science have long shaped the evolution of political–ecological thought. Decolonial approaches highlight how colonial legacies continue to structure environmental governance, frequently sidelining Indigenous knowledge systems and local ecological practices [19, 20, 85]. This theme reflects the increasing recognition of Indigenous environmental knowledge as both a scientific and political resource for sustainable governance.

#### Inclusion of Indigenous Peoples’ perspectives

Indigenous Peoples have long practiced adaptive environmental management grounded in observations of ecological dynamics [86]. Contemporary political ecology focuses on the struggles and social and environmental movements of Indigenous Peoples and calls for decolonisation, providing theoretical critiques of ecologies, science, practice, governance, and conservation [87, 88]. Political ecology contends that the exclusion and/or marginalisation of Indigenous Peoples perspectives and ontologies from environmental policy has perpetuated colonial power structures and environmental degradation [89, 90]. Indigenous knowledge provides ecological insights, weather forecasting techniques, sustainable agricultural practices, and adaptation strategies and is important for resilience [91]. For example, Inuit knowledge of sea ice patterns, animal behaviour, ecological changes, and weather patterns enables safer travel and hunting and adaptation to climate change impacts in the Arctic [91–93].

Political ecology advocates for the integration of such knowledge into mainstream governance frameworks, challenging the positivist assumptions that often dismiss local ecological expertise. The collaboration and knowledge co-production with Indigenous Peoples has challenged the dominant western discourses rooted in colonial approaches by incorporating Indigenous knowledge into research, policy, and governance frameworks [86, 94]. This approach highlights Indigenous philosophies on future planning and prediction [95]. While Indigenous methods effectively address environmental changes, efforts to align them with Western standards can undermine their value and hinder collaboration in environmental management [95, 96].

Political ecology posits that colonial and economic exploitation systems have historically shaped environmental governance that prioritises resource extraction for private accumulation and profit [97, 98]. This resulted in the dispossession of Indigenous Peoples from their lands and imposed colonial management and governance models that marginalised Indigenous knowledge systems [99, 100]. Despite growing recognition, Indigenous knowledge systems frequently encounter institutional barriers. Conservation projects in the Amazon, for instance, have displaced Indigenous communities under the guise of biodiversity protection—a phenomenon known as “green grabbing” [97]. This exclusion perpetuates colonial governance models that prioritize global ecological goals over local socio-ecological realities [101].

The exploitation of resources and marginalisation of Indigenous Peoples were integral to the development of global extraction industries, which treated labour and land as commodities to be controlled and exploited for profit [102–104]. Political ecology recognises that this legacy continues in contemporary environmental governance, where Indigenous Peoples are excluded from decision-making processes and Indigenous lands are appropriated by extractive industries for green grabbing [87, 97, 105], affecting Indigenous epistemologies, ontologies, and ways of life. This aligns with the interests of global capital, framing ecologies as resources for global benefit, neglecting the long-standing Indigenous sustainable practices [100].

The integration of Indigenous knowledge into climate governance — as in Inuit sea ice monitoring in the Arctic [91, 106, 107] — illustrates how epistemic diversity can directly enhance adaptation. However, “green grabbing” and market-based conservation schemes often appropriate Indigenous lands in the name of climate mitigation [108]. This underscores the need for political ecology to move beyond metaphorical calls for decolonisation towards material redistribution, land restitution, and sovereignty in climate policy.

#### Indigenous knowledge as resistance to economic exploitation

Colonial and capitalist systems have historically exploited both natural resources and Indigenous labour to fuel economic growth [43, 103]. Political ecology critiques this extractionist logic, highlighting how land dispossession and ecological degradation continue to disproportionately affect Indigenous territories [100]. For instance, rotational farming techniques practiced in the Andes demonstrate an ecologically sustainable alternative to industrial agriculture's monoculture model [109]. The decolonial turn in political ecology calls for epistemic justice by advocating for knowledge co-production between Indigenous communities and researchers [88]. This collaborative approach fosters context-specific governance strategies that align ecological sustainability with social justice imperatives.

Indigenous knowledge systems play an important role in challenging the exploitative and profit-driven logic of environmental exploitation [19]. They are based on principles of reciprocity, community bonds, and long-term sustainability, contrasting with economic development models that emphasise large-scale profits and resource extraction [110, 111]. Indigenous Peoples have long engaged in resource management practices grounded in an understanding of the interconnectedness of ecosystems, promoting ecological resilience in ways that industrial resource extraction fails to achieve [112, 113]. Political ecologists have documented how Indigenous knowledge in regions such as the Arctic, the Amazon, and the Andes offers sustainable alternatives to the dominant extractive industries and recognises the impact of European and North American colonialism that must be addressed by decolonising the research process and knowledge [91, 114–118]. Indigenous agricultural and land management practices—such as rotational farming, controlled burning, and communal land stewardship—not only prove more sustainable but also resist the commodification of nature that drives environmental exploitation [109, 119]. However, these practices are often marginalised by state actors and international organisations that prioritise large-scale industrial agriculture and conservation projects, further entrenching economic systems that profit from the exploitation of natural resources [120].

Political ecology analyses the limitations in top-down conservation policies that often impose external conceptions of “sustainability” and “nature” upon Indigenous Peoples [121, 122]. For example, conservation of biodiversity initiatives in the Amazon has led to the displacement of Indigenous Peoples under the pretext of forest protection, leading to socio-economic marginalisation, poverty, and environmental injustice [101, 123, 124]. The marginalisation of Indigenous voices in environmental governance aligns with the objectives of global capital, which considers Indigenous territories as commodities for resource extraction or mitigation of climate change impacts [97, 125]. Political ecology critically analyses this exclusionary model, highlighting the need for a decolonial approach that honours Indigenous rights, sovereignty, and control over productive resources. It acknowledges the historical and cultural significance of Indigenous environmental management practices and advocates for their present implementation for long-term environmental and resource sustainability. Political ecology aims to dismantle the colonial legacies embedded in contemporary environmental governance systems through decolonisation, fostering a more just and inclusive form of environmental governance and management.

Political ecology challenges the socioeconomic and political structures that prioritise profit and private accumulation of property over long-term sustainability and asserts that integrating Indigenous knowledge into environmental governance is an ecological necessity [126]. It emphasises Indigenous governance practices, community-based resource management, and collective land ownership as viable alternatives to extractive and exploitative economic systems [72, 127]. This underlines that nature should be seen as an integral part of societal well-being rather than as a commodity to be exploited.

### Climate change, justice, and vulnerability

#### Climate justice, vulnerability and socio-ecological dynamics

The climate crisis has intensified socio-environmental inequalities, with vulnerable communities experiencing disproportionate impacts despite minimal contributions to greenhouse gas emissions [128].). Political ecology's engagement with climate justice therefore examines the socio-political factors that shape climate vulnerability, while also acknowledging the ecological processes that mediate these vulnerabilities [129]. Climate justice, in this context, extends beyond distributive concerns about who bears the costs and benefits of climate impacts,it also interrogates procedural justice regarding who participates in climate governance and recognition justice concerning whose knowledge and experiences are valued [3, 128].

Political ecology highlights the disproportionate impacts of climate change on marginalised communities, who bear the least responsibility for greenhouse gas emissions, but their greatest impacts [130, 131]. It asserts that wealthy nations and corporations bear the primary responsibility for historical greenhouse gas emissions and continue to derive substantial profits from the fossil fuel economy [132, 133]. Whereas vulnerable communities in the “Global South” and low-income regions of the Global North disproportionately experience the impacts of climate change [134–136]. The concept of climate justice within the political ecology underscores the inequitable distribution of climate risk and vulnerability [137]. This approach emphasises that socioeconomic and political structures that prioritise profit over sustainability exacerbate climate change impacts experienced by marginalised groups [138, 139]. Political ecology emphasises that climate vulnerability is deeply influenced by underlying power structures and socioeconomic inequalities that make certain populations more susceptible to climate risks [129, 140, 141].

Socio-ecological dynamics in climate justice are deeply entwined with historical processes of colonisation, capitalist expansion, and environmental transformation [142]. The extraction of fossil fuels, deforestation for plantation economies, and the establishment of carbon-intensive infrastructure have disproportionately affected communities in the “Global South” while enriching industrial centers in the Global North [143, 144]. The resulting disparities in climate vulnerability cannot be understood through biophysical exposure alone,they are rooted in structural inequalities that shape adaptive capacities and access to resources. Political ecology provides a critical framework to analyse these dynamics, highlighting the interactions between ecological feedback mechanisms—such as changing rainfall patterns and rising sea levels—and the socio-political structures that determine resilience and vulnerability [145–147].

Political ecologists have documented that climate-induced flooding and heatwaves disproportionately affect low-income communities in informal settlements in cities of the “Global South” such as Dhaka, Mumbai, Chennai, Bengaluru, and Lahore, which is a result of social, economic, and political vulnerabilities [148–150]. The communities living in these settlements often lack the necessary resources and political power to influence policies and governance decisions, making them more susceptible to disasters [151]. This vulnerability is not incidental but the result of long-term neglect by international institutions and state actors that prioritise economic growth and profit over equitable urban development.

The global climate justice movement in political ecology has emerged as an important force calling for equitable approaches to addressing climate change [152]. This movement includes environmental justice activists, Indigenous Peoples, grassroots organisations, and labour unions, calling for a radical transformation of the global economy to address social inequality and climate change [153, 154]. It demands the redistribution of resources from wealthy nations and corporations to the communities most affected by climate change, particularly those in the “Global South” and Indigenous territories, who have contributed the least to global warming but face the greatest climate risks [128, 155].

A just transition to a low-carbon economy is crucial for addressing climate impacts in ways that ensure the benefits of decarbonisation are shared equitably across communities [156]. This ensures that the costs of the transition are not borne by marginalised communities or working class [157, 158]. Political ecology contends that a just transition necessitates a radical restructuring of the global economy that prioritises public investment, collective well-being, and democratic sovereignty over natural resources [159].

Scholars such as Mills-Novoa et al. [160] and Goldman et al. [161, 162] have shown how adaptation policies can inadvertently reinforce inequities when they fail to address power relations. Nightingale’s [79] work demonstrates that vulnerability is not simply exposure to hazards, but a relational condition produced through political-economic structures and historical dispossession. Anchoring these debates in concrete cases — from Dhaka’s informal settlements to Louisiana’s Indigenous coastal communities — reveals how colonial histories, extractive economies, and ecological degradation intersect to produce climate injustice [163, 164]. Political ecology here moves beyond technical adaptation planning to demand structural change: just transitions away from fossil fuels, redistribution of resources, and climate finance that addresses historical responsibility.

Political ecology has long intersected with environmental justice scholarship, and recent work has strengthened these connections by showing how both fields illuminate the production and experience of environmental inequalities [19]. While political ecology offers a structural analysis of power, political economy, and the historical drivers of socio-ecological harm, environmental justice foregrounds recognition, participation, and the uneven distribution of risks and benefits [4, 165]. Integrating these perspectives expands the analytical and epistemic tools available for understanding injustice, particularly through calls for epistemic diversification within justice scholarship [166]. Recent contributions demonstrate how political ecology’s attention to structural drivers can complement environmental justice’s focus on lived experience, highlighting how climate impacts, pollution burdens, and resource dispossession are shaped simultaneously by global political-economic processes and localised marginalisation [167–169]. Critical environmental justice scholarship is also broadening its scope to new domains such as illegal wildlife trade and conservation governance [170], while political ecologists are examining multispecies and posthuman forms of justice that extend beyond the human [171].

Together, these dialogues underscore the value of bringing political ecology’s structural critique into conversation with environmental justice’s normative and movement-oriented commitments—particularly in the context of deepening climate crises.

#### Vulnerability and inequality in climate change impacts

Climate vulnerability is not merely a function of biophysical exposure but is deeply rooted in historical patterns of exploitation, marginalisation, and uneven development [141]. Vulnerability is socially produced through historical land dispossession, labour exploitation, and governance failures, which intersect with dynamic ecological processes to shape localised climate risks [172, 173]. Political ecology, therefore, emphasises the importance of examining vulnerability as a relational phenomenon—one that arises from the interactions between socio-economic systems and environmental processes [174, 175].

For instance, informal settlements in Dhaka, Bangladesh, face recurrent flooding due to a confluence of ecological factors, such as riverine dynamics and urban heat island effects, compounded by political neglect and inadequate infrastructure [149]. The city's urban expansion has encroached upon wetlands that previously served as natural flood buffers [176], illustrating how decisions driven by land commodification and speculative real estate practices exacerbate ecological risks. The inhabitants of these settlements, primarily low-income migrants, contribute minimally to global carbon emissions yet experience some of the most severe climate impacts [164, 177]. In Mozambique, tropical cyclones Idai and Kenneth devastated coastal regions, exposing the compounded vulnerabilities created by poverty, colonial legacies, and state-centered governance models that excluded local communities from disaster planning [178]. The cyclones' impacts were magnified by mangrove degradation—an ecological process influenced by unregulated timber extraction and agricultural expansion [179, 180]. Here, political ecology reveals how climate-induced hazards intersect with socio-economic marginalisation and ecological mismanagement to produce climate injustices.

In coastal Louisiana, United States, Indigenous communities such as the Isle de Jean Charles Band of Biloxi-Chitimacha-Choctaw have faced progressive land loss due to rising sea levels, oil and gas extraction, and sediment starvation caused by river management projects [181, 182]. Despite official relocation programs, community members have encountered bureaucratic barriers, cultural dislocation, and the erasure of traditional ecological knowledge [183]. This case underscores the entanglement of environmental change, historical injustices, and contemporary governance failures in shaping vulnerability.

Political ecology contributes to climate justice debates by emphasising the socio-political determinants of vulnerability, such as land tenure insecurity, uneven access to adaptation resources, and exclusionary governance practices [184, 185]. By situating vulnerability within broader historical and ecological contexts, political ecology challenges technocratic approaches that treat climate risks as isolated phenomena divorced from societal structures [147, 173].

Political ecology draws attention to the disproportionate contributions of industrialised nations to greenhouse gas emissions and the uneven distribution of climate impacts across geographical and socio-economic lines. The principle of "common but differentiated responsibilities" (CBDR) underpins international climate negotiations, yet the operationalisation of this principle often neglects the historical processes of resource extraction and environmental degradation that have shaped present-day vulnerabilities [186–188]. For instance, the Caribbean island of Dominica, despite contributing negligible emissions, faces increasingly frequent and severe hurricanes, such as Hurricane Maria in 2017, which devastated 90% of the country's infrastructure [13]. Political ecologists argue that climate finance mechanisms must address these historical injustices by prioritising adaptation support for countries disproportionately affected by the colonial legacies of plantation economies and resource extraction.

The Sahel region in Africa illustrates the legacy of environmental and socio-economic disruption linked to colonial agricultural policies and contemporary climate change [9]. The degradation of the region's drylands has been exacerbated by policies promoting monoculture cash crops and excluding traditional pastoralist practices [189, 190]. Political ecology foregrounds how these historical legacies intersect with climate change to produce vulnerabilities.

Environmental degradation is not merely a consequence of mismanagement or technological insufficiency but is deeply rooted in the structural imperatives of economic systems that prioritise capital accumulation over ecological sustainability [191, 192]. Historically, resource extraction and environmental transformation have been driven by a logic of commodification, where forests become timber reserves, rivers become hydroelectric assets, and landscapes are reconfigured to serve market demands [193, 194]. This transformation abstracts ecosystems into units of exchange, erasing the complex, interdependent relationships that sustain ecological integrity. As land, labour, and natural resources are relentlessly integrated into global markets, socio-ecological disruptions intensify, disproportionately affecting communities whose livelihoods are intricately tied to local environmental conditions [195–197]. Political ecology, in this sense, interrogates how these systemic forces perpetuate environmental injustices, recognising that ecological crises are often symptomatic of broader socio-economic configurations that subordinate ecological well-being to profit imperatives.

#### Economic drivers and structural solutions to climate adaptation

In response to the climate crisis, international organisations and state actors have formulated a variety of climate adaptation policies aimed at mitigating vulnerability [198]. However, these policies are frequently influenced by the same economic interests that contribute to environmental degradation [199]. Market-based solutions—such as carbon trading, resilience investments, and green technology—predominate in the realm of climate adaptation, yet they often benefit affluent nations and corporations while failing to address the fundamental causes of climate vulnerability [200, 201]. Political ecologists critique these market-based approaches as superficial remedies that perpetuate the underlying economic structures of exploitation [202]. For instance, carbon markets commodify carbon emissions, enabling wealthy corporations to purchase carbon credits while continuing to pollute [203]. These markets transfer the costs of climate adaptation onto vulnerable communities, rather than holding the most polluting industries accountable for the damage they inflict [204]. This approach prioritises short-term profit over long-term sustainability and overlooks the structural inequalities that exacerbate climate vulnerability (Table 1).

**Table 1:** Key themes and processes in political ecology

Theme	Key processes
Posthumanism and more-than-human governance	Recognising non-human agency; analysing multispecies entanglements; examining how ecological processes (wildlife, water flows, pathogens, atmospheric systems) shape governance and unsettle human-centred decision-making
Decoloniality and Indigenous knowledge systems	Challenging colonial epistemologies; foregrounding Indigenous cosmologies, land relations, and governance systems; exposing epistemic injustice and advocating for pluriversal environmental futures
Climate justice and socio-ecological inequalities	Tracing uneven climate vulnerabilities; analysing how power, race, class, and colonial histories shape exposure, resilience, and adaptation; connecting local climate impacts to global political–economic systems
Market-based adaptation mechanisms and financialised climate governance	Examining carbon markets, offset schemes, biodiversity credits, and climate finance; analysing dispossession, enclosure, and uneven benefits under green capitalism; interrogating the commodification of nature
Historical legacies, colonial extraction, and vulnerability	Documenting how histories of extraction, land appropriation, and development interventions produce contemporary socio-ecological vulnerabilities and path-dependent climate risks
Technological and digital transformations in environmental governance	Analysing algorithmic risk production, satellite-based monitoring, AI-mediated climate decision-making, and digital data infrastructures; assessing how these technologies reproduce or challenge existing power relations
State power, territorialisation, and conservation regimes	Analysing how states use conservation, carbon forestry, and land-use zoning to assert authority; examining “political forests” and the territorialisation of space through environmental governance

Carbon offset projects, which frequently involve the establishment of monoculture tree plantations in the “Global South”, are often portrayed as “sustainable” solutions to climate change [205]. However, these projects often displace local communities, degrade biodiversity, and undermine the resilience of ecosystems [206]. Political ecology contends that authentic climate adaptation must transcend market-based mechanisms and focus on addressing the structural inequalities that render certain populations more vulnerable to climate impacts [138].

To effectively address the inequalities in climate vulnerability, adaptation policies must be designed to confront the structural drivers of climate change [207]. Mainstream climate adaptation efforts often emphasise technocratic solutions—such as improving flood defences, upgrading infrastructure, or implementing early warning systems [208, 209]. While these measures are important, they are insufficient if they do not address the underlying political and economic systems that perpetuate vulnerability [210]. Participatory, community-driven approaches are increasingly recognised as essential for effective and equitable climate adaptation [211]. These strategies must prioritise the needs and knowledge of marginalised communities rather than being dictated by external actors who prioritise economic growth and profit. For instance, urban climate adaptation plans should include residents from informal settlements in decision-making processes to ensure that their voices are heard and that policies are designed to address their specific vulnerabilities [212–214].

The interplay between climate change and wider political, economic, and historical forces has been richly documented, offering insights that deepen contemporary understandings of climatic change [215]. Recent scholarship also interrogates mitigation and adaptation efforts—ranging from mineral extraction and resource frontiers to the governance of climate-induced migration—showing how vulnerability is produced through policy design and uneven power relations [41, 216, 217]. Political ecology challenges the authority of powerful actors, question dominant discourses, and seek to understand how political, economic, and social factors influence the management, use, and distribution of natural resources, as well as the impacts of these processes on human communities and ecosystems [3, 218].

The concept of “othering” is explored by political ecology in connection with the production of the current climate crisis [219], shedding light on the neoliberal and colonial structures of domination that bear significant responsibility for our present crisis. Political ecology has been applied in the realms of colonialism and subaltern studies [220], biopolitics and racism [74], coloniality of climate change governance and climate refugees [216], and development studies [221]. Its analytical framework has been employed to assess the socioenvironmental implications associated with the expansion of renewable energy frontiers [222, 223], the rebranding of large hydropower projects as “climate-friendly” development [224], the conflicts and phenomena of “green grabbing” associated with wind energy generation [225, 226], the concept of “solar colonialism” [227], and the utilization of climate mitigation funds to promote biofuel plantations, as well as the creation of extractive landscapes through various forms of physical and structural violence [228].

Market-based adaptation soultions, such as carbon trading, carbon offset projects, and climate-resilient infrastructure investments, have gained prominence in global climate governance [203, 229]. These mechanisms often rely on neoliberal principles that commodify nature, treating carbon, water, and biodiversity as tradable assets [206, 230]. Political ecologists have critically analysed these approaches, revealing how market-based adaptation can perpetuate socio-environmental injustices by externalising costs to vulnerable populations and prioritising profit over ecological resilience [231, 232]. For example, carbon offset initiatives in East Africa have displaced local populations to establish monoculture plantations, disrupting ecological balance and community livelihoods [205]. In Kenya's Mau Forest Complex, communities that have historically relied on the forest for subsistence were forcibly evicted to accommodate carbon sequestration projects backed by international investors [233, 234]. While these projects contribute to corporate carbon neutrality claims, they disrupt local ecosystems by replacing biodiverse forests with eucalyptus plantations, which deplete water tables and degrade soil fertility [235, 236]. Political ecology critiques such initiatives for obscuring the social and ecological costs of offset schemes behind abstract carbon metrics.

In Brazil, the proliferation of REDD + (Reducing Emissions from Deforestation and Forest Degradation) projects has similarly sparked concerns regarding land tenure conflicts and environmental governance [143, 237]. Indigenous communities in the Amazon have reported cases where forested territories were appropriated by private actors seeking carbon credits, sidelining traditional stewardship practices that had preserved the forest for generations [118, 238]. The commodification of carbon, in this case, has transformed forests into profit-generating assets while disempowering the communities with the most intimate ecological knowledge of these landscapes [239–241].

Political ecology has long shown that carbon forestry and conservation programmes are also instruments through which states territorialise authority and reorder socio-ecological relations. Classic work on ‘political forests’ illustrates how state power is consolidated through mapping, enclosure, and the reclassification of land and livelihoods [242–244]. These processes intersect with broader neoliberal strategies that commodify nature—through carbon markets, offset schemes, and performance-based payments—transforming forests into calculable assets within global climate governance [245, 246]. More-than-human political ecologists remind us that forests, species, and ecological processes actively shape these interventions, often exceeding or disrupting state and market logics [247, 248]. Attending to these dynamics foregrounds how carbon forestry operates simultaneously as a state project, a neoliberal environmental instrument, and a multispecies assemblage that co-produces uneven climate governance outcomes.

Climate-resilient infrastructure projects, such as seawalls and flood control systems, often reflect broader socio-economic priorities that marginalise vulnerable populations [249, 250]. In Jakarta, Indonesia, the construction of the "Giant Sea Wall" was initially promoted as a defense against rising sea levels [251]. However, political ecology research revealed that the project primarily benefited affluent waterfront developments while displacing low-income fishing communities who rely on coastal ecosystems for their livelihoods [252, 253]. This case illustrates how adaptation investments, when guided by market logics rather than inclusive governance, can exacerbate rather than mitigate climate injustices.

Political ecology, therefore, questions the efficacy and equity of market-based adaptation by examining how such mechanisms redistribute climate risks and adaptation burdens along existing lines of social inequality. It challenges the assumption that market rationalities can inherently deliver climate justice, emphasising the need for adaptive strategies grounded in social equity, ecological integrity, and participatory governance.

Political ecology analyses the human dimensions of climate change to understand human capacities, resilience, adaptation, vulnerability, responses, and disparities in power dynamics inherent in the understanding and governance of nature [147, 254]. This includes scrutinising the politics of knowledge that favour Western scientific perspectives while marginalising Indigenous knowledge and local land-use practices [162]. It advocated for the importance of foregrounding colonialism in the examination of human vulnerability and adaptation to climate change and the framing of Indigenous Peoples and communities in terms of the local and the traditional [255]. Drawing on a deep appreciation for historical context, unequal political economies, and intricate ecological systems, political ecologists have actively criticised the frequently apolitical framings of climate change and how it is known and unevenly experienced [256–258].

#### Anthropocene and capitalocene

The concept of the Anthropocene—proposing that human activity has become a dominant geological force—has generated substantial debate within political ecology [259–261]. While the term has circulated widely in scientific, policy, and public arenas, political ecologists caution that its universalist framing risks depoliticising the socio-ecological crises it seeks to describe [35, 259]. By attributing responsibility to “humanity” in the abstract, the Anthropocene narrative obscures stark asymmetries in the production of environmental harm, masking the disproportionate roles of carbon-intensive industries, corporate actors, and affluent states [213, 262]. Such framings can inadvertently naturalise global inequalities by presenting planetary degradation as a collective human achievement rather than a historically uneven and politically mediated process.

In response, scholars have advanced the notion of the Capitalocene, a term that redirects analytical attention from humanity as a whole to the capitalist relations, extractive infrastructures, and imperial ecologies that underpin contemporary environmental crises [263, 264]. This reframing foregrounds how accumulation, commodification, and global market integration fundamentally structure ecological transformations, demanding governance models that address the systemic drivers of degradation rather than its symptoms [265, 266]. It also highlights the historical geographies of exploitation—slavery, colonial extraction, plantation agriculture, and fossil-fuel industrialisation—that have bound ecological change to racialised and uneven regimes of power.

Political ecology contributes to this debate by clarifying the entanglements between political economy and ecological change. Classic and contemporary work demonstrates how capitalist processes reconfigure both nature and society through expansion, enclosure, and intensified resource extraction [267, 268]. These analyses underscore that global environmental change is not merely an outcome of aggregate human activity, but a product of specific political-economic systems, institutional arrangements, and histories of domination. A Capitalocene lens thus offers a more nuanced account of environmental degradation and its uneven burdens, aligning with political ecology’s commitment to justice-oriented, relational, and historically grounded explanations of socio-ecological transformation.

## Future directions in political ecology

The future of political ecology lies in its ability to address the unprecedented socio-environmental challenges of the climate crisis while dismantling the historical and structural inequalities that shape vulnerability. This requires treating climate justice not as an adjunct theme, but as the organising principle that links political ecology’s core concerns with power, inequality, and ecology.

A critical first step is deepening the decolonisation of political ecology. While early works — such as those by Escobar, Hecht, and Bryant — engaged with colonial legacies [37, 142, 221], the urgency of the climate crisis demands a shift from acknowledging these legacies to materially transforming governance structures, redistributing resources, and restoring sovereignty to Indigenous and marginalised peoples. This entails moving beyond rhetorical inclusion of Indigenous knowledge towards long-term, equitable partnerships where communities set research agendas, define success, and hold decision-making power [50, 269].

The future direction of political ecology holds significant importance for global environmental justice movements, policy, research, and advocacy. The world is currently facing unprecedented socioenvironmental challenges. This has resulted from generations of fossil fuel use, industrial development, pollution, and resource degradation, leading to a wide range of environmental changes [270, 271]. These changes have cascading effects and can push the Earth towards a global ecological tipping point [28, 272]. These issues are happening within the current global political climate characterised by increased populism, authoritarianism, nationalism, climate change, injustice, and social conflicts [273, 274], which political ecology should focus on.

While political ecology has traditionally focused on the relationship between nature and society, there is a need to explore the intersectionality of various social categories, such as gender, race, class, and ethnicity, within its framework – crucially, this should be applied to knowledge production [275]. Though political ecology has been appreciated as a conceptual focus for lively debates, it has received criticism for the fact that Anglo-American contributions to it have been highlighted, leading to calls for political ecologies from around the world, rather than a singular political ecology [276].

Political ecology can further its own development by paying attention to ontologies and epistemologies suppressed by the mainstream – there are calls for this [277], which link this with the need to further political ecology’s decolonization. Indeed, the charge of Eurocentrism is one that political ecology has faced [278]. On the other hand, theorists of political ecology are debating whether and how the ostensible incommensurability of non-modern and Western ontologies within political ecology can be problematized and perhaps reconciled [87].

Political ecology can sometimes be critically examined as a colonial discipline due to its frequent reliance on Western academic frameworks, which often impose external perspectives on local environments and social structures, particularly in the “Global South” [169]. Historically, the field has sometimes perpetuated power imbalances by prioritising the concerns of global elites over Indigenous knowledge systems [279]. The utilisation of colonial-era data and methodologies has significantly influenced the study and understanding of landscapes and resource management practices [280]. The discipline’s emphasis on environmental degradation has at times, obscured the colonial exploitation that contributed to such degradation [281].

This highlights the imperative to decolonise political ecology by incorporating local voices and epistemologies. To achieve this, the discipline must promote collaboration among diverse knowledge systems. This entails not just acknowledging and appreciating Indigenous knowledge and local knowledge systems, which frequently originate from longstanding connections with the environment [269] but also utilising them to address socioecological problems. By integrating multiple knowledge systems, diverse ontologies, and knowledge co-production, political ecology can adopt a more comprehensive and inclusive approach to foster a broader and more holistic understanding of environmental challenges [282].

Decolonisation in political ecology involves addressing historical injustices and the impacts of colonialism [283]. This necessitates re-evaluating the concept of sovereignty and actively involving marginalised populations in the decision-making process. The call for decolonization should not be just limited to acknowledging some historic exploitation, but the one that transcends metaphorical gestures, is more than a lip service, and calls for returning the land and agency to the colonised populations around the world [19]. A crucial aspect of decolonization is shifting the power dynamics inherent in traditional research practices. Empowering marginalised communities to actively participate in research processes is central to decolonization efforts [284]. Participatory research approaches, which involve involving community members in the design, implementation, and dissemination of research [285], can challenge the hegemony of academic researchers and foster a more equitable knowledge co-production process. By embracing participatory research, political ecology can move beyond the extractive and exploitative research practices that have perpetuated colonial legacies.

In the context of decolonising political ecology, epistemological decolonisation must also be included in the future direction of not just decolonising who is adding to knowledge in the field but also how that knowledge is gathered. Existing epistemologies and research methodologies are too narrowly based on European and North American experiences, hindering their ability to capture non-European realities [286]. The Western scientific tradition, rooted in colonial conquest, exploitation, and slavery, needs decolonization to better understand and analyse the entire world [287]. The inadequacy of Eurocentric epistemic resources leads to hermeneutical injustice, which is an irreducible form of epistemic oppression. Addressing this oppression requires looking beyond the Eurocentric paradigm, as it may fail to recognise and address the underlying issues [288]. To dismantle the hegemony of Western knowledge, nondominant knowledge and its epistemes must be taken seriously [289]. Epistemic decolonisation could either complete the anti-colonial struggle or facilitate self-re-discovery for formerly colonised and oppressed communities [290].

Understanding the relationship between political ecology and environmental justice movements can help understand how grassroots activism, social movements, and advocacy efforts contribute to challenging power structures and promoting more equitable and sustainable socio-environmental outcomes. There have been calls for an insurrectionary political ecology which explores just these themes and for cross-fertilization between concepts derived from environmental justice approaches and political ecology [5]

Analysing the role of global governance institutions, such as international treaties, agreements, and organisations, in shaping socio-environmental processes can provide insights into the effectiveness of existing governance mechanisms and the potential for transformative change on a global scale. Indeed, there has been research that has taken this direction, such as for example, a study of the global political ecology of the Clean Development Mechanism [291, 292]. Understanding the intersections between political ecology and the United Nations Sustainable Development Goals can help identify synergies and tensions between socio-environmental objectives, and research in this direction is already visible [293]. The examination of environmental imaginaries and the governance of commodified nature should also be the focus of political ecology. Integrating environmental imaginaries into the analysis can enhance our understanding of how people perceive and construct their environment. Beyond evaluating environmental deterioration and risk, political ecology should explore the governance of commodified nature by thoroughly analysing practices such as environmental restoration, infrastructural development, and conservation.

While recent syntheses of political ecology [3, 9, 218, 256, 294, 295] map the field’s established terrain, rapidly shifting socio-ecological conditions indicate several emerging directions that remain under-theorised. One concerns the rise of anticipatory climate governance—forecasting models, early-warning systems, and predictive analytics that increasingly shape interventions before crises unfold [296, 297]. Political ecology could offer vital insight into how these predictive infrastructures redistribute uncertainty, responsibility, and risk. A second frontier lies in the growing entanglement of climate change with security institutions, humanitarian infrastructures, and border regimes, where ‘climate security’ narratives justify militarised responses to migration and environmental disruption [298, 299]. Finally, earth-systems governance and planetary boundaries frameworks are reshaping global environmental authority in ways that demand political–ecological critique, particularly regarding who defines thresholds, whose knowledges count, and how global limits are imposed locally [300–302]. These directions move beyond existing overviews by highlighting domains where political ecology can make novel conceptual and empirical contributions as climate governance becomes increasingly anticipatory, securitised, and planetary in scope.

The rapid advancement of technology, such as remote sensing, artificial intelligence, biotechnology, big data analytics, and robotics, presents new opportunities and challenges in the study of political ecology. Understanding their implications for power dynamics and resource control, how these technologies can be effectively integrated into political ecology research, and their implications for understanding and addressing socio-environmental issues is important for advancing the field [215, 303]. Speculative and experimental political ecologies are emerging sub-disciplines, in which technology or design approaches are used to model or create various possible future scenarios overlaid with concerns derived from the field of political ecology [304–306], allowing for decision-making to be steered towards equitable outcomes. More generally, exploring innovative research methods and approaches within political ecology, such as participatory mapping, citizen science, knowledge co-production, and collaborative research, can enhance the inclusivity, validity, and impact of research outcomes. Investigating the strengths and limitations of these methods can contribute to methodological advancements in political ecology research.

Technological innovations—from AI-driven climate modelling to remote sensing—offer new tools for political ecology, but must be deployed critically, particularly as digital and data-driven forms of environmental governance—including algorithmic risk assessments, satellite-based monitoring, and AI-mediated decision-making—can reproduce or challenge existing power relations. Community-led approaches such as participatory mapping, citizen science, and collaborative modelling remain vital for enhancing local agency in climate governance, especially when embedded within decolonial research frameworks that foreground place-based expertise [282, 285]. These emerging socio-technical terrains underscore the need to interrogate not only what technologies reveal, but also whose interests they serve and whose voices they exclude.

Finally, the field must maintain a dual focus: resisting the commodification of climate solutions while building alternative economies of care, reciprocity, and regeneration. This means challenging carbon market schemes, “green” extractivism, and solar/wind colonialism [108, 231], and amplifying community-led just transition movements. These futures are already being enacted — from Sámi resistance to wind power encroachment in Norway, to Andean Indigenous-led water governance — offering concrete pathways for political ecology to both study and support transformative climate action.

## Conclusion

Political ecology remains indispensable for confronting the climate crisis because it situates environmental change within the histories, structures, and power relations that produce vulnerability and resilience. By tracing these dynamics across scales — from local adaptation struggles to global political economies — political ecology offers analytical tools that climate policy and science alone cannot provide. Perhaps the greatest challenge that lies ahead for political ecology is epistemic decolonisation; this would entail going beyond the inclusion of diverse voices to the inclusion of diverse ways of thinking and knowing. Political ecology holds a great promise for understanding contemporary challenges, what causes them, their historical roots, and ways of addressing them. But at the same time, it needs self-reflection in terms of methodological approaches and the dominance of certain perspectives. It needs to foster the solidarity and collaboration of colonised and marginalised peoples and their ways of life to empower its methodological and conceptual base. Its engagement with climate justice, particularly through distributive, procedural, and recognition lenses, remains central to its future relevance. Political ecology’s strength will lie in its capacity to integrate historical political economy, more-than-human relations, and decolonial practice into coherent strategies for climate justice — ensuring that the fight against climate change is inseparable from the struggle for a more equitable and ecologically viable world.

## Data Availability

No datasets were generated or analysed during the current study.
